# Novel circulating tumor cell-based blood test for the assessment of PD-L1 protein expression in treatment-naïve, newly diagnosed patients with non-small cell lung cancer

**DOI:** 10.1007/s00262-019-02344-6

**Published:** 2019-05-14

**Authors:** Yen-Lin Chen, Wen-Chien Huang, Feng-Ming Lin, Huangpin B. Hsieh, Chia-Hsun Hsieh, Ruey Kuen Hsieh, Kuo-Wei Chen, Ming-Hong Yen, James Lee, Stephen Su, Twinkal Marfatia, Shih-En Chang, Padma Sundar, Bruce Patterson, Drew Watson, Rui Mei, Manana Javey

**Affiliations:** 1 Cardinal Tien Hospital, New Taipei, Taiwan; 2 Mackay Memorial Hospital, Taipei, Taiwan; 3 CellMax Life, Sunnyvale, CA USA; 4 Chang Gung Memorial Hospital, Taoyuan, Taiwan; 5 Cheng Hsin General Hospital, Taipei, Taiwan; 6 Cathay General Hospital, Taipei, Taiwan; 7 University of California, San Francisco, San Francisco, CA USA; 8 IncellDx, Menlo Park, CA USA

**Keywords:** PD-L1 expression, Non-small cell lung cancer, Circulating tumor cells, Liquid biopsy, Checkpoint inhibitor therapy

## Abstract

We evaluated the analytical and clinical performance of a novel circulating tumor cell (CTC)-based blood test for determination of programmed death ligand 1 (PD-L1) protein expression status in real time in treatment-naïve non-small cell lung cancer (NSCLC) patients. CTCs were detected in 86% of patients with NSCLC (I–IV) at the time of diagnosis, with a 67% PD-L1 positivity rate (≥ 1 PDL + CTC). Among 33 NSCLC patients with PD-L1 results available via both tissue immunohistochemistry (IHC) and CTC assays, 78.9% were positive according to both methods. The CTC test identified an additional ten cases that were positive for PD-L1 expression but that tested negative via IHC analysis. Detection of higher PD-L1 expression on CTCs compared to that in the corresponding tissue was concordant with data obtained using other platforms in previously treated patients. The concordance in PD-L1 expression between tissue and CTCs was approximately 57%, which is higher than that reported by others. In summary, evaluation of PD-L1 protein expression status on CTCs isolated from NSCLC patients is feasible. PD-L1 expression status on CTCs can be determined serially during the disease course, thus overcoming the myriad challenges associated with tissue analysis.

## Introduction

For newly diagnosed patients with advanced non-small cell lung cancer (NSCLC), the national guidelines recommend comprehensive genomic profiling for targeted therapy selection and testing for programmed death ligand 1 (PD-L1) protein expression in tumor tissue for benefit assessment of immune checkpoint inhibitor (ICI) therapy [1].

ICIs targeting the PD-1/PD-L1 pathway have become part of the standard of care management for NSCLC patients, and several antibodies have been approved by the Food and Drug Administration (FDA) in the first- and second-line settings.

In clinical studies, progression-free survival (PFS) and overall survival (OS) upon ICI treatment were greater in NSCLC patients with high PD-L1 expression in tumors [2, 3]. However, only a small subset of the patients responded to treatment, whereas patients with low or no PD-L1 expression in tumors also responded to treatment [2–4]. Therefore, it is challenging but critical to stratify NSCLC patients for ICI risks and benefits.

Several companion diagnostic (CD) PD-L1 tests have been developed and approved by the FDA. These tests evaluate PD-L1 expression by utilizing immunohistochemistry (IHC) analysis of tumor tissue obtained at the time of diagnosis. Despite FDA approval, there is no well-standardized approach across even the IHC CD PD-L1 tests, and the PD-L1 expression cutoffs and testing standards are widely variable across the antibody clones and devices utilized.

Furthermore, IHC-based PD-L1 CD assays face tissue availability issues and challenges due to biological phenomena, such as tumor evolution, tumor heterogeneity, variable PD-L1 protein expression and protein expression fluctuation over the course of treatment [5–7].

Tumors evolve over the course of the disease, thus limiting the utility of the IHC PD-L1 test as the only tool for ICI risk/benefit assessment administered at a single time point in the disease course. In addition to tumor evolution, PD-L1 protein expression fluctuates over the course of treatment and displays variable expression across the tumor tissue, which is not fully represented in small biopsy specimens due to sampling bias [5–7].

To overcome the above noted tissue-based testing-related issues, several “liquid biopsies” have been evaluated for prediction of ICI benefits. One of the approaches is to assess PD-L1 protein expression on circulating tumor cells (CTCs). CTCs are derived from primary and metastatic tumor lesions and are shed into the peripheral circulation [8–10]. CTCs alone have been associated with poor prognosis in NSCLC patients [11, 12]. In addition, monitoring of PD-L1 protein expression levels on CTCs may potentially provide useful information about the PD-1/PD-L1 pathway inhibition status during the disease course.

Thus far, to the best of our knowledge, three main studies have been conducted evaluating PD-L1 expression on CTCs from NSCLC patients upon ICI therapy [11–13]. These studies utilized different CTC detection platforms, namely, Cell Search, ISET and Epic Sciences [11–13]. These platforms for CTC isolation are based on fundamentally different principles, which may lead to different CTC detection sensitivities and specificities in NSCLS patients. Moreover, two of the three studies enrolled heavily pretreated NSCLC patients, and only one study enrolled treatment-naïve patients. Based on previously published data, PD-L1 expression fluctuates upon treatment, and previously treated patients may have altered expression of PD-L1 on CTCs [5–7]. To understand the true baseline PD-L1 expression pattern on CTCs, it is important to evaluate “treatment-naïve”, newly diagnosed NSCLC patients. Here, we wished to evaluate PD-L1 expression on CTCs detected in blood from newly diagnosed, treatment-naïve NSCLC patients utilizing the highly sensitive CellMax (CMx) microfluidic CTC detection platform. We also aimed to establish the concordance between CTC and tumor tissue PD-L1 protein expression and finally compare the results to data previously published for treated and treatment-naïve NSCLC patients.

## Materials and methods

### CTC PD-L1 assay development

Anti-PD-L1 antibody, clone 28.8 (BioINK, directly conjugated to Alexa flour 647, IncellDx, Menlo Park, CA), was used to develop the CTC assay for PD-L1 expression status assessment. First, the anti-PD-L1 antibody was titrated on the manufacturer-provided positive and negative control cell lines according to the instructions. In addition to the manufacturer’s recommended controls, clone 28.8 was tested on ten cancer cell lines: three breast cancer (T47D, SK-BR3, MDA-MB-468), three lung cancer (H1975, H661, H520), two colorectal cancer (HT29, HCT-116) and two prostate cancer (PC3, LnCaP) cell lines. The cell lines were stained and compared with the controls supplied by the manufacturer. Contrived samples were prepared by spiking approximately 200 cells of each cell line into 2 mL of peripheral blood with preservative, and the spiked samples were run through the proprietary CMx microfluidic chip. The released cells were then stained with antibodies against cytokeratin 18 (CK18-ab133263, AbCAM, Cambridge, UK), CD45 (F10-89-4, AbCAM, Cambridge, UK) and PD-L1 (BioInk, IncellDx, Menlo Park, CA, USA), followed by staining with fluorophore-conjugated secondary antibody [Goat anti-Rabbit IgG (H + L) Cross-Adsorbed Secondary Antibody, Alexa Fluor 568 (A-11011, ThermoFisher, for CK18) and Goat anti-Mouse IgG2a Cross-Adsorbed Secondary Antibody, Alexa Fluor 488 (A-21131, ThermoFisher, for CD45)] and then nuclear counterstaining with DAPI (4′,6-diamidino-2-phenylindole) to identify cancer cells for enumeration.

### Patient samples

Upon written consent, 2 mL of peripheral blood from 51 NSCLC patients (*n* = 51) was processed for analysis of PD-L1 expression on CTCs. Among the 51 subjects, 24 were diagnosed with stages I and II NSCLC, and 23 were diagnosed with late-stages III and IV disease. Staging information was not available for four patients. All patients were treatment-naïve at the time of blood draw. Sex was evenly distributed among the subjects, with 24 males and 27 females, and age ranged from 37 to 84, with a median age of 64 (Table 1).

**Table 1:** NSCLC patient characteristics

Stage		Age	Sex (M, F)
Unknown	4	46–64	1 M, 3 F
Stage I	18	48–82	10 M, 8 F
Stage II	6	53–78	3 M, 3 F
Stage III	7	59–75	2 M, 5 F
Stage IV	16	37–84	8 M, 8 F

**Fig. 1:**
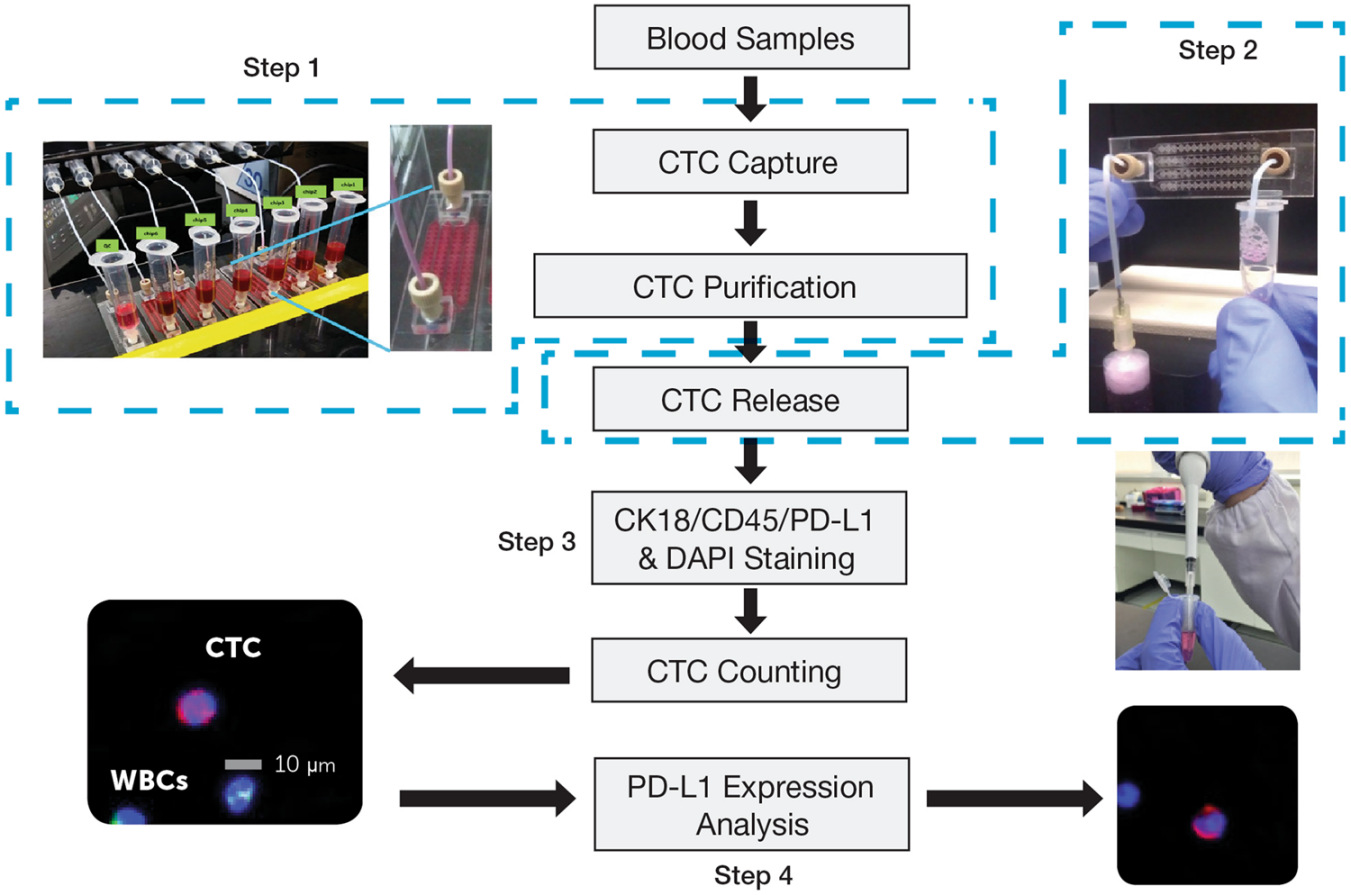
Workflow for CTC capture and PD-L1 analysis

The CMx CTC PD-L1 assay was performed on the CMx™ microfluidic platform (workflow depicted in Fig. 1). Briefly, 2 mL peripheral blood was run through a CellMax microfluidic chip, and CTCs were captured with a proprietary EpCAM (epithelial cell adhesion molecule) antibody implanted on an anti-fouling lipid bilayer coating, which promotes increased capture sensitivity and specificity as described in a previous publication. CTC purification was accomplished by a gentle in-chip wash with phosphate-buffer saline (PBS). Captured cells were then released from the chip through a sweep of property-matched air foams that separate the lipid bilayer from the chip surface, avoiding cell damage due to harsh breakage of antigen–antibody bonds. Released cells were transferred to a 10-mm, circular membrane and stained with antibodies against CK18, CD45, and PD-L1 and with DAPI counterstain for CTC enumeration and PD-L1 expression analysis.

Using a Leica DM6B automatic fluorescence microscope and Leica’s LAS-X automated image acquisition software suite, 100 (10 × 10) 16-bit monochrome images were acquired (by raster scan) per membrane in each of the 4 channels (Alexa Fluor 568 for CK18, Alexa Fluor 488 for CD45, Alexa Fluor 647 and DAPI for nuclear staining) with a 10 × (NA0.32) Leica objective. The 10 × 10 images in each channel were stitched and together covered a square that showed a 10-mm diameter membrane area.

For CTC screening, MetaMorph software (Molecular Device, San Jose, CA, USA) was used with a set of criteria for IF intensity cutoff and contrast in all three channels. Candidate cells were reviewed using the proprietary software CTC Reviewer V2.0 (CellMax, Sunnyvale, CA, USA) with a set of criteria based on IF intensity cutoff for each marker, as well as cyto-morphology and a set of rules for white blood cell (WBC) exclusion. Each selected CTC met the following criteria: (1) Cell size ≥ 9 μm with intact cellular morphology, (2) CK18 staining intensity > cutoff, (3) CD45 staining intensity < cutoff, (4) no typical WBC nuclear morphology, (5) the IF intensity cutoff for PD-L1 positivity was established at an IF intensity cutoff value, which consisted of contributions from the average intensities of the negative control and fluorescence background, and (6) complete circumferential or partial linear plasma membrane PD-L1 staining.

**Fig. 2:**
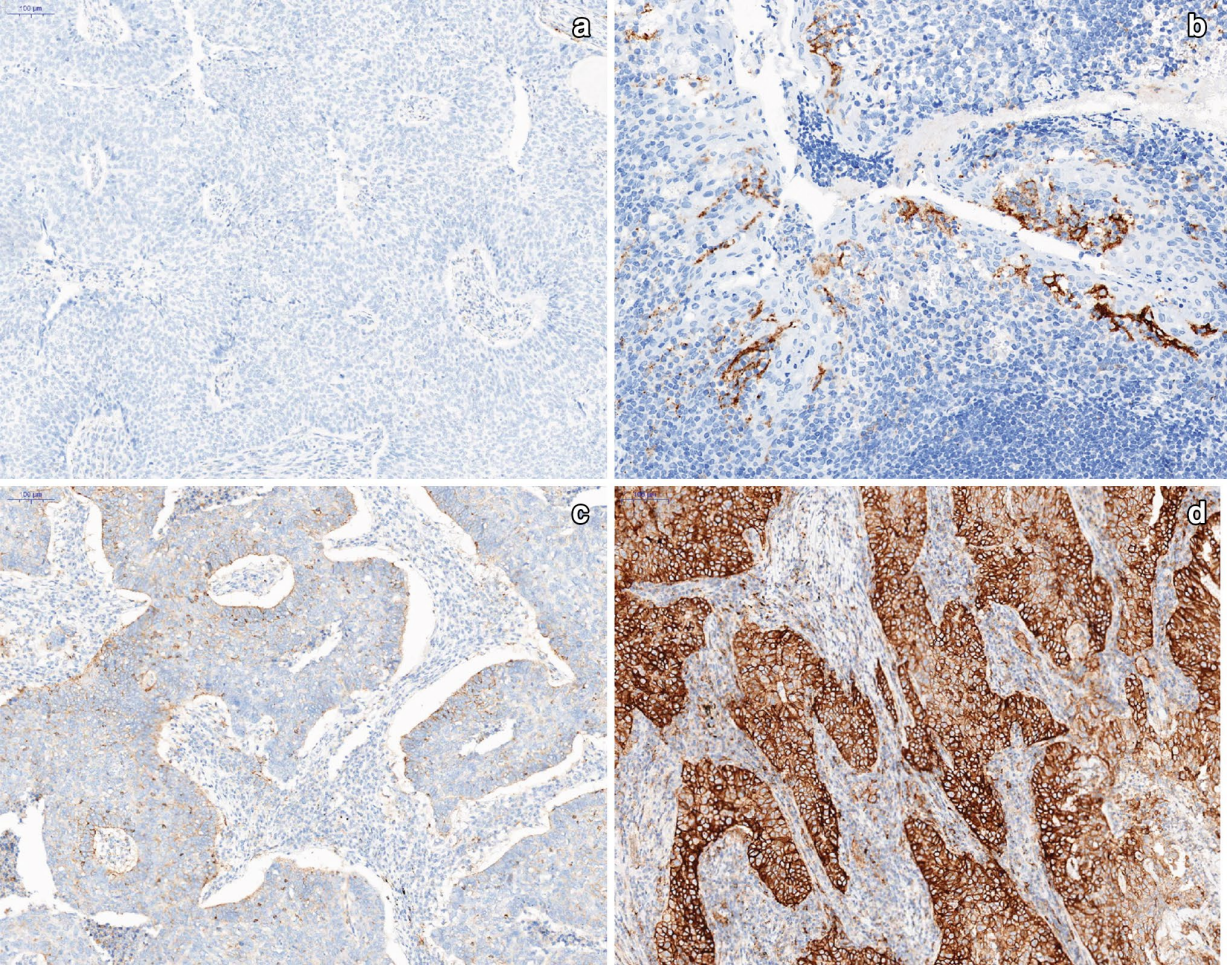
Examples of PD-L1 IHC staining of FFPE sections from NSCLC patients (× 100): **a** PD-L1(−) negative; **b** positive control (× 200), **c** PD-L1(+) positive, < 50% immunoreactive, **d** PD-L1(+) positive, > 50% immunoreactive

PD-L1 expression in tumor tissue was assessed in formalin-fixed, paraffin-embedded (FFPE) tissue sections (3-μm thick) and analyzed by the practicing anatomic pathologist. Briefly, tissue sections on glass slides (Mutokagaku, Japan) were preheated prior to IHC staining (Fig. 2). Upon deparaffinization and antigen retrieval, PD-L1 IHC staining was conducted using the 22C3 clone (22C3 PharmDx Kit, DAKO) on a Ventana BenchMark XT instrument, and the tissue sections were counterstained with hematoxylin. Finally, the slides were washed, dehydrated in a series of ethanol and xylene solutions, and cover slipped. The PD-L1 protein Tumor Proportion Score (TPS) was assessed on an Olympus BX41 microscope, based on partial or complete staining (≥ 1%) relative to all viable tumor cells. For each case, the TPS was assessed using at least 100 viable cells in the specimen, and an interpretation was made as to whether the sample exhibited no PD-L1 expression (< 1%) or any PD-L1 expression (≥ 1%).

## Results

To confirm the analytical validity of PD-L1 IF staining of CTCs, ten cancer cell lines with known PD-L1 expression levels (Genevestigator, transcriptome database) were utilized, including three lung cancer cell lines with high (H1975), intermediate (H661) and low (H520) expression levels.

**Fig. 3:**
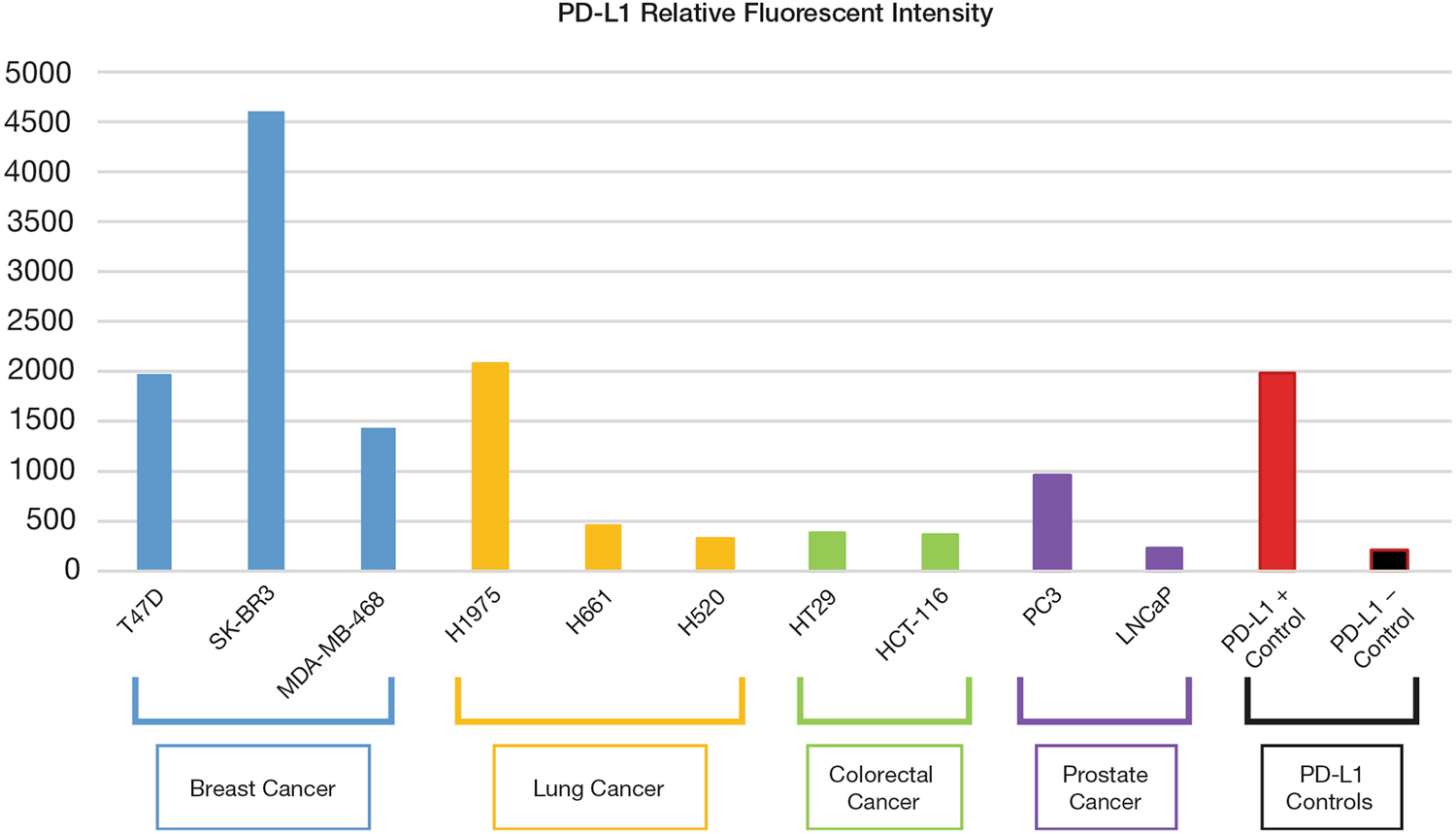
PD-L1 expression levels in ten cancer cell lines and in PD-L1 positive and negative control cells from the BioINK staining kit

All three positive lung cancer cell lines showed higher immunofluorescence (IF) staining intensity than the IF cutoffs established based on negative controls. Overall, the relative fluorescence intensities of these cancer cell lines, as calculated by the average of the IF stained cells, showed a trend consistent with the transcriptome database, confirming binding specificity of the PD-L1 antibody. Among these cell lines, the high PD-L1-expressing SK-BR3 cell line showed a greater than 20-fold difference in IF intensity compared with the PD-L1-negative control cells, indicating a dynamic range of 20 × for the fluorescence assay and assuring sensitive detection of CTCs that could exhibit low to high levels of PD-L1 expression (Fig. 3).

**Fig. 4:**
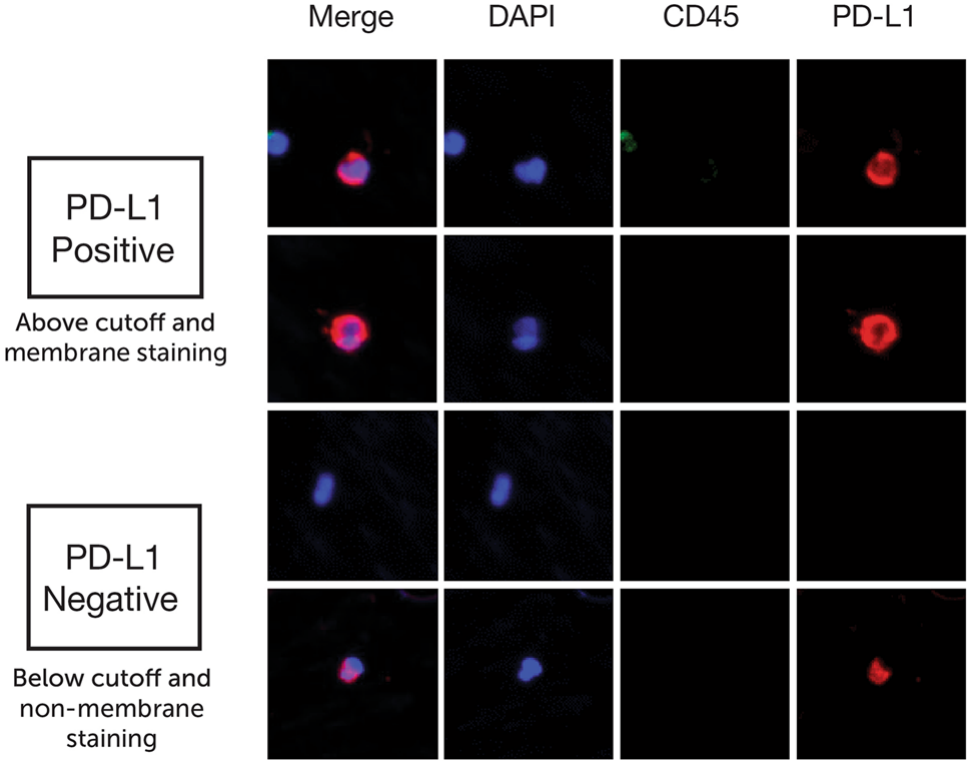
Example images of PD-L1 positive and negative staining patterns

PD-L1 protein expression was defined as the percentage of positive membranous staining. The latter was defined as complete circumferential or partial linear plasma membrane staining. As shown in the example images (Fig. 4) for PD-L1 immunofluorescence staining, PD-L1(+) staining was defined as an average PD-L1 intensity above the cutoff (which was determined by summing the negative control and background intensities) with visibly stronger complete circumferential or partial linear plasma membrane staining than cytoplasmic staining.

For two patients, blood was unavailable for testing. Among the remaining 49 NSCLC patients, CTCs were detected in 86% (42/49) of patients (average of 6 CTCs and a range of 0–47 CTCs), including 88% (21/24) of early stage patients, 87% (20/23) of late-stage patients (Table 2) and 1 of 2 patients with unknown stage; 67% (28/42) of patients with detectable CTCs were found to have at least one PD-L1(+) CTC.

**Table 2:** CTC detection rate and PD-L1% in treatment-naïve NSCLC patients

	All patients	Early stage (stages 1–2)	Advanced stage (stages 3–4)	Unknown stage
Subject #	49	24	23	2
CTC detection (≥1 CTC/2 mL)	42 (86%)	21 (88%)	20 (87%)	1 (50%)
PD-L1(+) CTC	28 (67%)	12 (57%)	16 (80%)	0
PD-L1(−) CTC	14 (33%)	9 (43%)	4 (20%)	1 (100%)

Thirty-three NSCLC patients with available tumor tissue and detectable CTCs were used to establish concordance between the two methods. The resulting 2 × 2 confusion matrix is shown in Table 3. Nearly, 80% of PD-L1(+) patients according to IHC were also CTC PD-L1(+), with one or more PDL1(+) CTCs. PD-L1 CTC testing using the IF method identified an additional ten (*n* = 10) cases with PD-L1(+) CTCs that had tested negative in the IHC assay. Previously, it has been shown that the PD-L1 positivity rate in CTCs can be twice that observed in tissue, potentially attributable to uneven distribution of PD-L1 in tissue, which leads to false negatives [12]. Several of the samples had multiple PD-L1(+) CTCs. Four patients positive for PD-L1 protein expression in tissue tested negative for PD-L1 protein expression on CTCs. Only one (1) CTC was detected in peripheral blood from three of these four (3/4) patients, and eight (8) CTCs were detected in one (1/4) patient, but all were negative for PD-L1 protein expression. Three out of four (3/4) of these patients were low expressers based on IHC, with PD-L1 expression ranging from 8 to 10%, and one patient (1/4) exhibited 70% PD-L1 protein expression according to IHC. CTCs were not detected in seven (7) patients. Tissue was available from five of these seven (5/7) patients, four of which (4/5) demonstrated low PD-L1 expression ranging from 0 to 30% in tumor tissue based on IHC, while one out of five (1/5) showed 90% PD-L1 protein expression in the IHC analysis.

**Table 3:** 2 × 2 Confusion matrix and performance measures

CTC	IHC			
		PD-L1(+)	PD-L1(−)	Total
	PD-L1(+)	15	10	25
	PD-L1(−)	4	4	8
	Total	19	14	33

## Discussion

It is well accepted that cancer evolves during the disease course, especially under the influence of therapy. Hence, utilization of archival material for real-time assessment of the tumor profile may not accurately reflect the current PD-L1 protein expression status. The majority of PD-L1 expression studies that have been conducted utilized archival tumor tissue [5–7]. Given that PD-L1 protein expression is quite dynamic and can be affected by various anti-cancer regimens, the results obtained based on archival material in clinical studies may have led to inadequate patient stratification for ICI risks and benefits [5–7]. In addition, NSCLC diagnosis and treatment eligibility are often determined based on core needle biopsy (CNB) specimens. Small biopsies, such as CNBs, introduce sampling bias and may not fully reflect the heterogeneous tumor landscape, including variable PD-L1 expression across the tumor tissue. Finally, the tissue may not be available or too scant to triage for both molecular and PD-L1 status workup. Hence, increasing efforts have been made to develop blood-based assays to further stratify NSCLC patients for ICI risks and benefits in real time.

Several outcome studies have demonstrated the feasibility of determining PD-L1 protein expression on CTCs isolated from patients with NSCLC during ICI therapy [11–13].

The CTC isolation techniques utilized in the above-mentioned clinical studies included immune-magnetic capture with EpCAM enrichment (Cell Search), size-based filtration (ISET), and whole blood digital pathology [11–13]. Given that the principles of these technologies are fundamentally different, it is important to compare and contrast the baseline PD-L1 expression across the platforms in NSCLC patients. In addition, two of these three studies enrolled heavily pretreated NSCLC patients, which potentially may affect the PD-L1 expression pattern.

Nicolazzo et al. utilized a CellSearch device and reported a very high CTC detection rate at the baseline. Although the sample size was small (*n* = 24), their results were quite different from the results obtained by others utilizing the same CellSearch CTC detection platform with 40% sensitivity [11]. The authors themselves postulated that the latter was because their study cohort was composed of heavily pretreated patients with advanced NSCLC disease.

The study by Guibert et al. which utilized the ISET platform enrolled a higher number of previously treated patients (*n* = 96) and demonstrated a high CTC detection rate (93%) at the baseline [12].

Although Boffa et al. enrolled 112 treatment-naïve patients, only 26 patients (23%) had detectable CTCs (or “circulating cells associated with malignancy”, CCAM, as the authors called them). According to the authors, this represented only half of that reported for various IHC-based PD-L1 studies, and one of the possible reasons was limited assay sensitivity.

In our study, 86% of treatment-naïve NSCLC patients had detectable CTCs (≥ 1 CTC), among which 67% showed PD-L1 protein expression. This number is three times higher than the number of PD-L1-expressing CTCs reported by Boffa et al. in their treatment-naïve NSCLC study cohort [13]. Conversely, the two other studies observed much higher PD-L1 expression at the baseline in their pretreated patients, ranging from 83 to 95% [11, 12]. The PD-L1(+) CTC detection rate in previously treated NSCLC at the baseline appears to be similar between the two studies but significantly higher than that in naïve untreated patients assessed by us and Boffa et al. It is noteworthy to mention that our platform demonstrated a much higher PD-L1(+) CTC detection rate than the CTC detection method used by Boffa et al. This discrepancy could be due to lower sensitivity of the technology utilized by Boffa et al. [13].

Although we did not study PD-L1 expression during the ICI treatment course, others have observed that approximately half of patients had persistent PD-L1(+) CTCs after 6 months of therapy and this was associated with poor outcome and suggestive of a PD-L1 predictive role later in the course of treatment [11, 12].

In concordance with others, we also demonstrated that CTCs are more likely to be positive for PD-L1 than the corresponding tumor tissue [11, 12]. Among the three studies, only the study by Guibert et al. tried to establish concordance with the corresponding tumor tissue and showed ~ 45% concordance, while our study showed 57% concordance.

The CMx CTC platform has been previously clinically validated in colorectal cancer (CRC). In a study conducted with more than 700 patients, this platform demonstrated high sensitivity and specificity for detection of precancerous lesions and all stages of CRC [14, 15]. In the present study on NSCLC, the CMx CTC detection platform demonstrated a high CTC detection rate (86%) across all NSCLC stages. Downstream analysis of CTC PD-L1 expression assessment detected most (78.9%) tissue PD-L1(+) cases as well as 10 additional cases that were negative for PD-L1 in the IHC assay. This could be explained by the fact that CTCs represent heterogeneous tumors better than FFPE sections of excisional biopsy specimens and especially of CNB specimens due to tissue sampling bias. To establish concordance, each case was carefully examined for PD-L1 expression both in the tissue and on CTCs (≥ 1 CTC), and the concordance was approximately 57%.

Thus, based on previously published clinical data [11–13], we think that assessment of PD-L1 expression on CTCs may serve as an ancillary piece of information to stratify NSCLC patients for risk and benefits during ICI therapy. Additional outcome studies are planned to establish the role of the CMx PD-L1 CTC test in the clinical setting.

## Abbreviations

CD: Companion diagnostics
CK18: Cytokeratin 18
CMx: CellMax
CNB: Core needle biopsy
CTCs: Circulating tumor cells
ctDNA: Circulating tumor DNA
EpCAM: Epithelial cell adhesion molecule
FDA: Food and Drug Administration
FFPE: Formalin fixed paraffin embedded
ICI: Immune checkpoint inhibitors
IF: Immunofluorescence
IHC: Immunohistochemistry
NSCLC: Non-small cell lung cancer
OS: Overall survival
PD-L1: Programmed death ligand-1
PFS: Progression-free survival
